# Comparison of Two Risk Assessment Scores in Predicting Peri-Implantitis Occurrence during Implant Maintenance in Patients Treated for Periodontal Diseases: A Long-Term Retrospective Study

**DOI:** 10.3390/jcm11061720

**Published:** 2022-03-20

**Authors:** Amélie Sarbacher, Ioanna Papalou, Panagiota Vagia, Henri Tenenbaum, Olivier Huck, Jean-Luc Davideau

**Affiliations:** Department of Periodontology, Dental Faculty, University of Strasbourg, 8 Rue Saint-Elisabeth, 67000 Strasbourg, France; ame.sarbacher@hotmail.fr (A.S.); papalou.ioanna@gmail.com (I.P.); penyvagia@hotmail.com (P.V.); htenen@gmail.com (H.T.); huck.olivier@gmail.com (O.H.)

**Keywords:** risk assessment scores, peri-implantitis, risk factors, periodontal disease, maintenance

## Abstract

Background: There is a need for reliable risk assessment tools to better predict peri-implantitis occurrence. This study compared the long-term prognosis value of two models of risk assessment scoring in predicting peri-implantitis. Methods: Seventy-three patients with treated periodontitis representing 232 implants and attending long-term implant maintenance were evaluated. The Periodontal Risk Assessment (PRA) score, which combines only periodontal risk factors/indicators, and the Implant Risk Assessment (IRA) score, which combines both periodontal and implant risk factors/indicators, were calculated during implant maintenance. Peri-implantitis was defined by the presence of probing depth ≥6 mm with bleeding on probing/suppuration and bone level ≥3 mm. Analyses were performed at the patient level. Results: The mean implant follow-up was 6.5 years. Peri-implantitis incidence was 17.8%, and high-risk PRA and IRA percentages were 36.9% and 27.3%, respectively. High-risk PRA and IRA were significantly associated with peri-implantitis incidence, with hazard ratio (HR) = 4.8 and 3.65, respectively. Risk factors/indicators considered separately showed reduced associations with peri-implantitis. Conclusions: The PRA score combining periodontal parameters and IRA score combining both periodontal and implant parameters have comparable value in predicting peri-implantitis. These scores could allow practicians to intercept the risk of peri-implantitis and to manage follow-up modalities in patients with treated periodontitis.

## 1. Introduction

The prevention of peri-implantitis is considered as a growing issue regarding peri-implantitis occurrence, with functional and aesthetic consequences [1,2,3]. Peri-implantitis occurrence and severity are influenced by risk-factor distributions [1,4,5,6]. These risk factors include not only implant and prosthesis characteristics but also systemic and behavioral factors as well as those related to the periodontal environment [2]. Complex interactions between risk factors have been shown to amplify or reduce their prognosis value as demonstrated by the interaction between the type of implants (bone-level implants) and the over-contoured restoration [7,8]. The variability in diagnosis and the prognosis value of each risk factor, considered separately, suggest the importance of developing new clinical prognosis tools for day-to-day clinical practice [5,9,10,11,12].

Disease risk assessment scores combine various parameters with associated risk scales, leading to a unique and synthetic prognosis value for each patient and a patient-risk stratification at a given time of follow-up [10,13]. Therefore, they could help to optimize clinical decision making and improve oral health [14]. In periodontology, scoring tools, such as the Periodontal Risk Calculator (PRC) [15], Perio Risk [14], the Periodontal Risk Assessment (PRA) [16], and their modifications, have been developed and evaluated for a long time [13,17]. The reliability of each of these tools has been demonstrated for predicting tooth loss [14,17,18,19,20,21] and/or periodontitis recurrence/progression [20,22] during the long-term periodontal follow-up, and they are mainly used to adapt periodontal maintenance frequencies at the end of active periodontal therapy [13,22,23].

As dental implants also present risks of biological complications in the long term, the use of various risk assessment scores, including systemic/behavioral, periodontal, and also implant and prosthesis risk factors/indicators, has been also described, and they more or less demonstrate good performance in predicting the occurrence of peri-implantitis [11,12,24]. These scores could be useful for identifying and managing modifiable risk factors both after as well as prior to implant placement and prosthesis treatment, to adapt maintenance frequency, and to communicate the risk to the patient [10,11]. However, the relative heterogeneity of the parameters included to calculate risk scores raises the question of parameter selection criteria and calculation methods as previously discussed for periodontal diseases [17]. In some studies, only highly associated risk factors in the tested population are included in score calculation [11,12,25]. However, the choice of parameters could be based on the results of recent studies addressing risk factors/indicators of biological complications, such as in the design of the Implant Disease Risk Assessment (IDRA) score [10]. Therefore, the reliability of such prognosis tools may be influenced by a combination of risk factors/indicators and risk stratifications as well as the selected definition criteria of peri-implantitis (i.e., threshold values for probing depth and bone loss) as shown for peri-implantitis diagnosis [26].

A previous retrospective cohort study demonstrated that periodontal parameters evaluated at different time points of periodontal and implant follow-up differentially influence peri-implantitis incidence [6]. These data suggest that simplified and optimized prognosis tools could be helpful for maintaining and improving peri-implant tissue health. Therefore, the main purpose of this study was to evaluate and compare PRA with a risk assessment score that combines periodontal and implant and prosthesis risk factors/indicators in terms of their association with the incidence of peri-implantitis during long-term follow-up.

## 2. Materials and Methods

### 2.1. Studied Population

**Figure 1:**
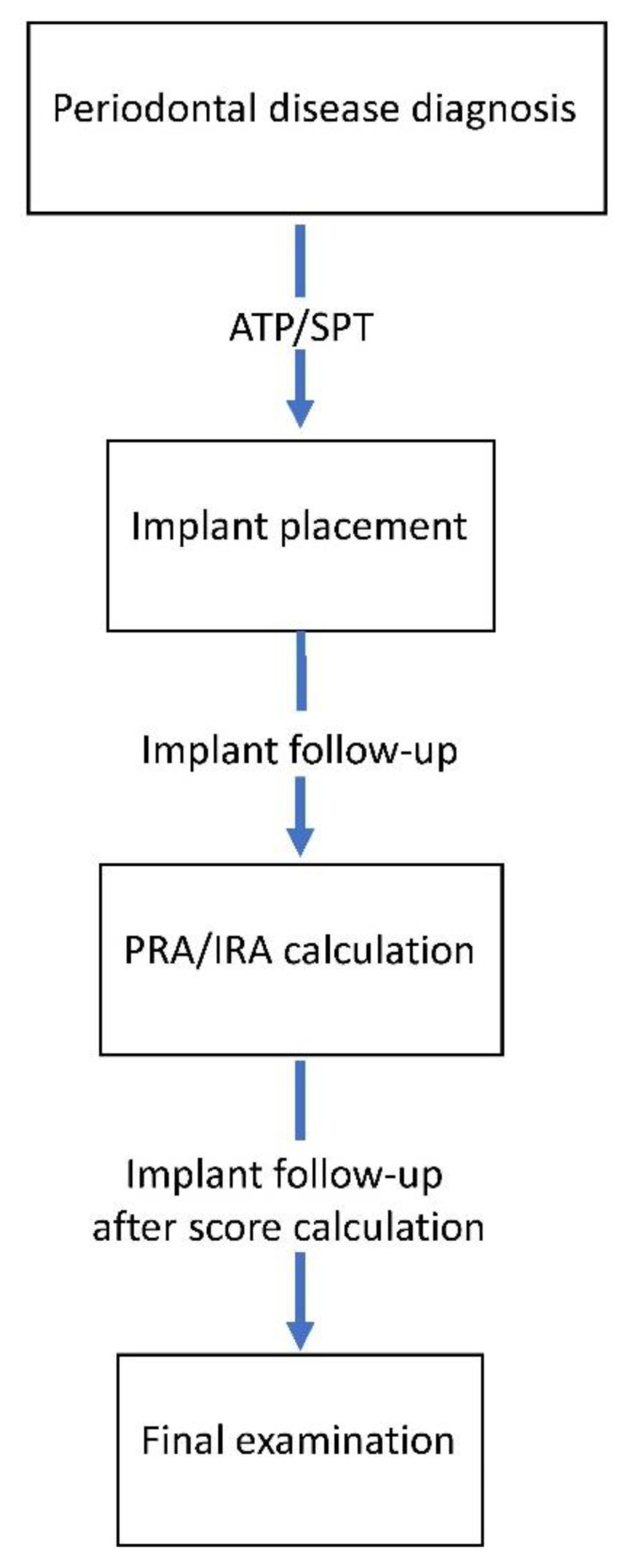
Sequence of periodontal and implant follow-up. APT/SPT, active and supporting periodontal therapies; PRA/IRA, Periodontal and Implant Risk Assessment scores.

The Ethical Committee of Strasbourg University Hospital approved the present retrospective cohort study (AMK/BG/2016-95—ClinicalTrials.gov ID: NCT03841656). All subjects were informed about the objectives of the study and provided their informed written consent for involvement. The study was conducted according to the principles stated in the Declaration of Helsinki (2013) [27]. To be included in this study, patient should have the following: (a) one or more implants placed at the Department of Periodontology, University Hospital, Strasbourg; (b) initial periodontal diagnosis and active and supporting periodontal therapy performed at the same Department of Periodontology before implant placement; (c) available updated demographic, medical, periodontal, and implant-related data during follow-up; (d) at least twelve residual teeth at the day of implant placement; and (e) a risk assessment score calculated at least 3 years or earlier before the final examination. Patients in need of antibiotic prophylaxis for oral examination and treatments were excluded. The different steps of periodontal and implant follow-up are described in Figure 1.

Dentate adults who had undergone periodontal and implant treatment before 2017 at the Department of Periodontology were identified from the clinic database. Following the screening of all completed files, 175 patients who met the inclusion criteria were contacted by phone call and invited for an examination between September 2017 and December 2019. Among them, 50 patients could not be reached, while 52 patients were excluded either due to death or difficulty to attend the recall appointment (disease and relocation) or refusal to participate in the study. A total of 73 patients were available for a final clinical and radiographic examination.

### 2.2. Implant Follow-Up

A comprehensive clinical examination was performed at every maintenance visit. Regarding smoking, patients were divided in 3 groups: non-smokers (who have never smoked), former smokers (who quit >5 years ago before examination and score calculation), and current smokers (at least 1 cigarette a day). During follow-up, periodontal and implant examinations were performed by dental students under the supervision of experienced periodontists (H.T., O.H., J.-L.D.). Full-mouth periodontal pocket depth (PPD), bleeding on probing (BOP), and suppuration were recorded. All the measurements were performed at six points of each tooth and implant using a PCPUNC 15 probe (HuFriedy, Chicago, IL, USA). The periodontal reason for tooth loss (TL) during follow-up was determined according to the reason mentioned in the patient file or using periodontal charting and radiographs before tooth extraction [19,28].

In selected patients, 224 tissue-level and 8 bone-level implants (Straumann, AG^®^, Basel, Switzerland) were inserted at the Department of Periodontology in Strasbourg. Implants were placed at the end of active periodontal therapy or during supporting periodontal therapy. Periodontal and implant maintenance was provided at the Department of Periodontology in Strasbourg involving professional biofilm removal from teeth and implants [29]. At every recall visit/examination, all evident pathologic peri-implant conditions were recorded and treated according to implant maintenance protocol (Cumulative Interceptive Supportive Therapy—CIST) [30].

During follow-up, the frequency of recommended visits ranged from 3 to 6 months depending on periodontal and implant outcomes. Patients with no maintenance visits at the Department of Periodontology over a continuous 2-year period were defined as non-compliers [31,32].

### 2.3. Radiographic Analysis

A radiographic examination was performed on digital orthopantomography and peri-apical radiographs obtained using long cone parallel technique. Digital pictures of radiographs performed before 2016 have been obtained using video camera and analyzed using a single 27-inch HD monitor and ImageJ software (Wayne Rasband, National Institute of Health, Bethesda, MD, USA). Implant bone level (BLi) was measured on peri-apical radiographs as the distance from the junction between smooth and rough implant surfaces for tissue level implant or from implant fixture shoulder for bone-level implant to the first bone-to-implant contact on mesial and distal aspects of implant by two calibrated examiners (P.V., I.P.). The most elevated measurement (mesial or distal) was selected [33]. For BLi measurements, radiographic distortion was considered using the implant length as a reference. The periodontal bone loss in relation to patient age (BL/age) was estimated by orthopantomography, in which the worst affected site was considered [34].

### 2.4. PRA and IRA Calculations

PRA and IRA were calculated during implant follow-up after implant functional loading and at least 3 years before final examination. In case of multiple implants, the implant with the highest risk level among implants was selected to calculate the scores. The definitions of PRA and IRA are detailed in Table 1 and Table 2.

PRA calculation was based on six parameters as previously described: (a) percentage of BOP, (b) number of residual PPD ≥ 5 mm, (c) tooth loss due to periodontitis, (d) BL/age considering the worst site affected, (e) systemic (diabetes) or genetic factors, and (f) environmental factor (smoking status) [16,17,18].

IRA calculation used eight categories of parameters derived from IDRA definition [10]. IRA included some of the PRA parameters: percentage of BOP, number of residual PPD ≥ 5 mm, and BL/age. Other parameters were also considered: (a) history of stage II, III, and IV periodontitis; (b) compliance level; (c) periodontitis susceptibility based on periodontitis staging and grading [35]; (d) distance from restorative margin to marginal bone (RM-bone); and (e) prosthesis characteristics. However, some modifications of IDRA parameter definition and categorization have been made considering the periodontal status and compliance profile of the studied population. Indeed, a large majority of patients (84.93%) presented a high risk when using the original IDRA calculation, demonstrating that IDRA could not be used as is in the studied population.

History of stage II, III, and IV periodontitis was based on periodontal diagnosis established prior to periodontal treatment and was based on radiographic bone loss and/or PPD and/or clinical attachment level (CAL) according to the 2017 World Workshop on the Classification of Periodontal and Peri-Implant Diseases and Conditions [35]. Patients with an initial diagnosis of gingivitis or stage I periodontitis, defined by PPD < 5 mm and/or CAL < 3 mm and/or bone loss <15% of root length, were considered not to have a history of stage II, III, and IV periodontitis. Presence of history of stage II, III, and IV periodontitis was placed in moderate-risk category, and the high-risk category was not allocated.

Only two levels of compliance were used to determine patient adherence to supporting periodontal/implant therapy before score calculation. Compliance levels were based on compliance definition as previously described [31,32]. Non-compliance, defined as no maintenance visit for at least 2 years, was placed in the high-risk category.

Periodontitis staging and grading during implant follow-up was evaluated using clinical and radiographic data, as described in the implementation of the new classification of periodontal disease [36].

Tissue-level and bone-level types were only considered for RM-bone parameter categorization.

Low-risk plaque score was defined as having less than 4 sites around implant with a plaque index (PI) > 1 [37], with no poor fit and/or cement excess. High-risk plaque score was defined by the presence of more than 3 sites with PI > 1. Poor fitting was defined by radiographic evidence of an open margin between the abutment and restoration [38].

For risk assessment scores, low PRA and IRA patients have all parameters in the low-risk categories or only one in the moderate-risk category. Moderate PRA and IRA patients have at least two parameters in the moderate-risk category but at most one parameter in the high-risk category. A moderate IRA patient could also have one parameter in the high-risk category and all others in the low-risk category. High PRA and IRA patients have at least two parameters in the high-risk category.

### 2.5. Case Definition for Patients with Peri-Implantitis

Peri-implantitis definition was based on the 2017 World Workshop on the Classification of Periodontal and Peri-Implant Diseases and Conditions clinical case definition of peri-implantitis in the absence of initial data: probing depth ≥ 6 mm with BOP/suppuration and radiographic signs of bone level ≥ 3 mm apical to the most coronal portion of the intraosseous part of the implant [39].

### 2.6. Examiner Calibration for BLi Evaluation

Examiners underwent inter-examiner calibration on radiographs of ten patients with 38 implants. The percentage of agreement between the two examiners (I.P., P.V.) within ±0.5 mm for BLi was 83.78%. The intraclass correlation coefficients were >0.8.

### 2.7. Statistical Analysis

A patient was considered as a statistical unit. The occurrence of peri-implantitis during follow-up was defined as the primary outcome variable. Cumulative survival rates of peri-implantitis were calculated using Kaplan–Meier survival curves to consider the impact of implant follow-up time on peri-implantitis incidence. Receiver operating characteristic (ROC) curves were calculated to evaluate the discriminatory ability of PRA and IRA. Sensitivity, specificity, and positive and negative predictive values were also calculated. Cox regression analyses estimating hazard ratio (HR) with 95% confidence intervals were performed for each of the demographic, periodontal, and implant parameters and for the risk scores. Occurrence time of peri-implantitis was defined as the first occurrence of peri-implantitis per patient after PRA/IRA calculation. Cohen’s kappa coefficients were calculated to evaluate agreement between risk measured by the different risk scores. Logistic regression analyses were performed to evaluate the impact of PRA on TL per year (TL/Y) at final examination. For all regression analyses, patients with low and moderate PRA were gathered in one low/moderate group. Differences were considered as significant when *p* < 0.05. Analyses were performed using statistical software (XLSTAT, Addinsoft, Paris, France).

## 3. Results

### 3.1. Patient Characteristics at Final Examination and Peri-Implantitis Incidence

In the studied population at final examination, the mean age and number of males were 66.6 (±7.33) years and 33 (45.21%), respectively. In all, 36 patients (49.3%) were never smokers, 28 (38.3%) were former smokers, and 9 (12.3%) were current smokers. The duration of implant follow-up was 6.52 (±2.42) years after PRA/IRA calculation (range: 3 to 14 years). TL and TL/Y were 0.52 (±1.11) and 0.09 (±0.21).

### 3.2. Patient Characteristics at PRA/IRA Calculation Time

The mean age was 60.08 years at score-calculation time. The numbers of smokers and former smokers were 17 (23.29%) and 20 (27.40%). One patient was a heavy smoker. Three patients had controlled diabetes. Regarding periodontal parameters, the tooth loss was 8.44, the percentage of BOP was 24.29%, and 32.88% of patients presented > 25% BOP. The mean number of PPD ≥ 5 mm was 7.85. Furthermore, 30.41% and 32.88% of patients had more than eight and six sites with PPD ≥ 5 mm, respectively. The BL/age was 0.59, and 8.22% of patients had a BL/age > 1. A total of 65 (89.04%) patients had a history of stage II, III, and IV periodontitis, while 13 (17.81%), 50 (68.49%), 6 (8.22%), and 4 (5.48%) were classified Stage II—Grade A/B, Stage III—Grade A/B, Stage III—Grade C, and Stage IV, respectively. Sixty-one (83.56%) patients were considered compliant and twelve (16.43%) non-compliant. Seventy (95.89%) patients had only tissue-level implants. Forty-eight (65.75%) and three (4.11%) patients presented low-risk plaque scores and high-risk plaque scores, respectively, while twenty-two (30.14%) patients presented an implant with supramucosal poor fit (Table 3).

### 3.3. PRA and IRA Distribution

Most patients (52.05%) presented a moderate-risk PRA. High-risk PRA patients were 36.99%, while only 10.96% of patients presented a low risk. There were no low-risk IRA patients, moderate-risk IRA patients represented 53 (72.6%) of cases, and high-risk IRA patients represented 20 (27.4%) (Table 4). Concerning agreement between scores for risk evaluation, high-risk PRA and IRA matched in 16 patients (Cohen’s kappa coefficient 0.559).

### 3.4. Impact of PRA and IRA on Peri-Implantitis Incidence

During follow-up, peri-implantitis occurrence after PRA/IRA calculation was 13 (17.81%). Two implants were lost for peri-implantitis reason and were considered as peri-implantitis. No peri-implantitis was detected before PRA/IRA calculation.

**Figure 2:**
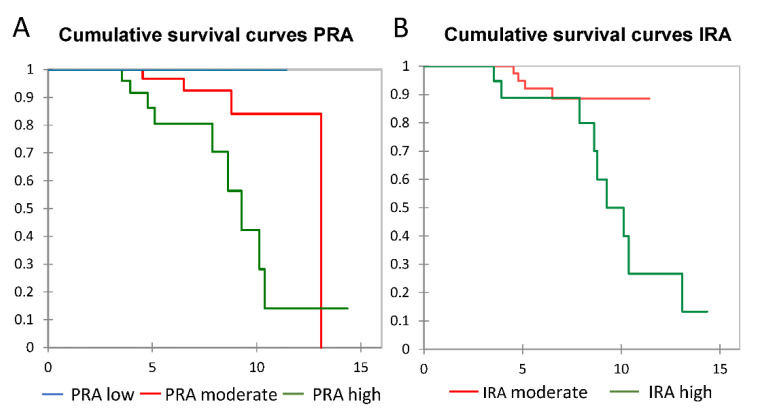
Kaplan–Meier curves for peri-implantitis risk score values determined by (**A**) PRA and (**B**) IRA. PRA, Periodontal Risk Assessment; IRA, Implant Risk Assessment.

Kaplan–Meier survival curves for peri-implantitis by PRA were significantly different with log-rank = 0.015. Comparison between PRA subgroups showed that moderate- and low-risk curves were not significantly different. A significant difference between curves for peri-implantitis was also observed for IRA with log-rank = 0.027 (Figure 2).

**Figure 3:**
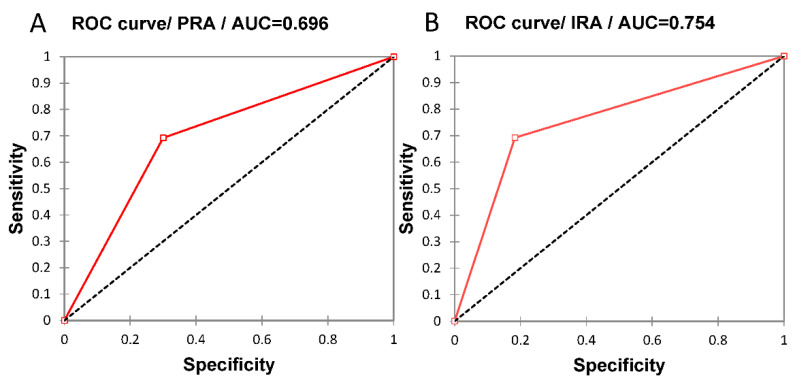
ROC curves illustrating the performance in predicting peri-implantitis occurrence in the case of risk scores calculated by (**A**) PRA and (**B**) IRA.

The areas under the ROC curve (AUCs) for PRA and IRA were 0.696 and 0.754 for peri-implantitis events, respectively (Figure 3). The sensitivity, specificity, positive predicting values, and negative predicting values were 0.692, 0.7, 0.333, and 0.913 for PRA and 0.692, 0.817, 0.450, and 0.925 for IRA.

Cox regression analyses were performed with high-risk PRA subgroups versus low/moderate-risk PRA regarding Kaplan–Meier survival curve distribution. Among parameters included in different risk scores, tooth loss > 8 was nearly significantly (*p* = 0.067) associated with peri-implantitis (HR = 3.07), and BL/age >1 was significantly (*p* = 0.003) associated with peri-implantitis (HR = 6.13). The other parameters were not found to be significantly associated with peri-implantitis. However, high periodontitis susceptibility was nearly significantly (*p* = 0.071) associated with peri-implantitis (HR = 3.086). Cox regression analyses showed that high-risk PRA patients were 4.7 times (HR = 4.78) more likely to have peri-implantitis during follow-up. High-risk IRA was also significantly associated with peri-implantitis incidence (HR = 3.65) (Table 5). Furthermore, during follow-up, in high-risk PRA patients, TL and TL per year of follow-up (TL/Y) were higher in high-risk PRA patients than in low/moderate-risk PRA patients: 0.81 versus 0.35 (*p*= 0.082) for TL and 0.15 versus 0.06 for TL/Y (*p* = 0.069).

## 4. Discussion

The present retrospective cohort study demonstrates that PRA based on periodontal parameters and IRA based on periodontal and implant prosthesis parameters allowed the identification of patients at high risk for peri-implantitis. PRA and IRA could be similarly predictive of peri-implantitis incidence in patients with treated periodontitis.

In the studied population, the percentage of high-risk PRA was 36.99% and appeared more or less comparable to percentages determined in other long-term periodontal follow-up studies, such as 30.6%/42.07% [18], 40% [23], 36.9% [22], 25.2% [40], and 28% [17]. The value for high-risk IRA patient percentage (27.39%) was lower but comparable to that of PRA. There were no low-risk patients according to IRA, while 10.96% of patients were at low risk according to PRA. This could be explained by the high percentage (89.04%) of patients with a history of stage II, III, and IV periodontitis. Indeed, in the study of De Ry et al. [24] on IDRA evaluation, only one patient was categorized as low risk, and 80% of 80 selected patients had a history of periodontitis based on the presence of bone loss. The agreement (Kappa coefficient = 0.559) between high-risk PRA and IRA scores was moderate [41], while the number and type of included parameters in each score differed. Tooth loss, smoking, and systemic status were separately considered to calculate PRA contrary to IRA, but these parameters were included in staging and grading of periodontitis to calculate IRA [35]. The influence of implant and prosthesis parameters in the determination of high-risk IRA compared to periodontal-related parameters appeared less pronounced. Indeed, the percentages of high-risk value of RM bone and prosthesis parameters were low, i.e., 4.11% of bone-level and/or high-risk plaque score implants. In the study of Petsos et al. [17], the agreement between periodontal scores PRA and PRC was low (Kappa coefficient = 0.23). PRC also included compliance and prosthesis parameters. However, in this later study, 30% of patients have prosthesis risk factors that could explain the higher impact of these factors on risk calculation.

PRA appeared highly predictive of peri-implantitis. Indeed, high-risk PRA patients were 4.7 times more likely than low/moderate-risk PRA patients to develop peri-implantitis during follow-up. PRA was classically associated with tooth loss (risk ratio = 2.67) [23] and periodontitis progression/recurrence (odds ratio (OR) = 5.79) [22] during periodontal maintenance. As expected, a nearly significant (*p* = 0.069) increase in the TL/Y rate was observed in high-risk PRA patients compared to low/moderate-risk PRA patients (0.15 versus 0.6). The fact that a periodontal risk assessment score had such a high prognosis value could be related to the impact of periodontal status on peri-implantitis incidence previously observed in a similar cohort [6]. Many studies investigating and comparing periodontal, implant, and prosthesis risk factors of peri-implantitis showed that the impact of periodontal status was one the most important factors [11,38,42,43]. Furthermore, some parameters included in PRA calculation, such as tooth loss, could indirectly reflect implant risk factors/indicators for peri-implantitis [44]. Indeed, tooth loss and the number of implants were correlated (data not shown), and the number of implants is considered to be a risk indicator of peri-implantitis occurrence in many studies [6,45,46].

IRA also had a high predictive value for peri-implantitis (HR = 3.9) lower than that of PRA. However, IRA performed better than PRA in predicting peri-implantitis occurrence in patients (AUC of 0.754 and 0.696, respectively). Both AUC values could be considered acceptable. PRA and IRA had the same sensitivity (0.692), but the specificity of IRA (0.817) was elevated and higher than for PRA (0.7), suggesting that IRA could better identify patients at risk of peri-implantitis. In the recent study of De Ry et al. [24] using the IDRA score, the odds ratio of high-risk IDRA patients having peri-implantitis was 2.67 but was not significant, and the AUC of IDRA appeared lower (0.613). In other studies, the AUC of the peri-implantitis risk assessment score combining periodontal and implant risk factors/indicators appeared to be better (or higher), such as 0.794 [11] and 0.858 [12]. These higher AUC values can be explained by the choice to only combine parameters individually associated with peri-implantitis occurrence in the studied populations. The choice of periodontal and implant parameters as well as their risk values used for IRA calculation were based on the results of recent studies on varied populations identifying general important risk factors/indicators associated with peri-implantitis occurrence [10], which could have limited their predictive performances. Furthermore, previous studies performed on similar cohorts have shown the impact of such risk factors/indicators on peri-implantitis occurrence [6] and peri-implant tissue conditions [47]. Interestingly, PRA and IRA parameters demonstrated few associations with peri-implantitis incidence when considered individually. Only periodontal BL/age > 1 appeared significantly predictive of peri-implantitis (HR = 6.13). In the study of De Ry et al. [24] using the IDRA score, the highest individual predicting performance was observed for BL/age (AUC = 0.739), while the other ones had AUC values close to IDRA AUC or lower. These data show that risk factors/indicators may have a higher predictive value in combination than when considered separately, as previously shown for tooth loss and/or periodontitis progression predictive value of PRA during periodontal maintenance [18,19,22].

PRA and IRA were not assessed systematically at a specific time of periodontal and implant follow-up, such as just after restoration delivery as shown for IDRA [24] or at one-year follow-up [12]. However, the change of patient-risk category throughout maintenance could be limited, as shown for PRA [18,22,40], and the presence of various follow-up periods after PRA/IRA calculation was specifically considered in Cox regression analyses. In patients with multiple implants, the worst value of RM bone and prosthesis has been used to calculate IRA at patient level, as initially proposed for IDRA [10]. However, peri-implantitis occurrence was determined on all the implants fulfilling inclusion conditions, as described in another study [12]. In the De Ry et al. study [24], only implants with the worst values were considered to evaluate the rate of peri-implantitis in contrast to the present study, suggesting that IDRA-predictive value was determined at implant level more so than patient level [24]. Implant/tooth/site-related risk factors/indicators, including the type of restoration material [12], BL/age [10,16], and presence of furcation [15], have been used in the calculation of different validated risk assessment scores at patient-level, suggesting that such local parameters could reliably reflect the overall peri-implantitis risk at patient level.

The present study has some potential limitations. The retrospective study design could lead to some misclassifications of exposures and outcomes. However, clinical procedures and examinations have been standardized and were supervised by experienced periodontologists, as described for the majority of such long-term follow-up studies [13]. Some adaptations of IDRA parameters have been made considering cohort specificity. The objective assessment of plaque accumulation was considered as a risk factor/indicator, as described in de Araújo Nobre et al. [12], and defined as the high-risk plaque score characteristic. In the study of De Ry et al. [24] using IDRA score, not cleanable was based on the expected risk of plaque accumulation due to restoration contour or implant location limiting access for the patient and clinician when attempting to clean the prosthesis. However, the impact of the not cleanable parameter greatly also depended on the ability of the patient to maintain efficient oral hygiene and to remove dental plaque. In the choice of compliance parameter risk scale in IRA, the impact on TL of various compliance definitions previously observed at the same department of periodontology [32] and in another study [31] was considered, and a long, continuous period without maintenance was selected to be a risk for peri-implantitis. In these studies, as also shown here, TL rates were comparable to TL rates observed in studies using a more restrictive definition of non-compliance [32]. Furthermore, there was no consensus regarding a minimal recall interval frequency to prevent peri-implantitis [48,49]. Finally, the low percentage or absence of low-risk PRA or IRA patients did not allow low-risk prognosis value evaluation. The specificities of the studied population indicated that results could not be directly generalized to other populations.

In conclusion, this study demonstrated that PRA/IRA calculation during periodontal and implant follow-up may be useful in predicting peri-implantitis occurrence and improving prevention of peri-implant diseases in day-to-day clinical practice. The similar PRA and IRA predictive values suggests that they similarly identified the high risk of developing peri-implantitis in patients with treated periodontitis. PRA and/or IRA may be used to adapt treatment modalities at different follow-up times.

## Figures and Tables

**Table 1:** PRA categories of risk and corresponding parameter values.

Score	PRA		
Parameters	Low Risk	Moderate Risk	High Risk
BOP%	0 to 9%	10 to 25%	>25%
Nb of PPD ≥ 5 mm	0 to 4	5 to 8	>8
BL/age	0 to 0.5	>0.5 to 1	>1
Tooth loss	0 to 4	5 to 8	>8
Systemic status	No	No	Yes
Smoking status	Non- and ex-smokers	Smoker < 20 cig/day	Heavy smoker > 19 cig/day

PRA, Periodontal Risk Assessment; BOP, bleeding on probing; PPD, probing pocket depth; BL/age, bone loss ratio/age of the worst site affected; Nb, number.

**Table 2:** IRA categories of risk and corresponding parameter values.

Score	IRA		
	Low Risk	Moderate Risk	High Risk
BOP%	0 to 9%	10 to 25%	>25%
Nb of PPD ≥ 5 mm	0 to 2	3 to 6	>6
BL/age	0 to 0.5	>0.5 to 1	>1
History of stage II, III, and IV periodontitis	No	Yes	
Periodontitis susceptibility	IA	II A/B, III A/B	III C, IV, IV C
Compliance level	Compliant		Non-compliant no maintenance visit for at least 2 years
RM-bone	Tissue level		<1.5 mm, bone level
Prosthesis/plaque score	Less than 4 sites with PI > 1	Poor fit—supramucosal	Poor fit—submucosal, cement excess, more than 3 sites with PI > 1

IRA, Implant Risk Assessment; RM, restoration margin; PI, Plaque Index.

**Table 3:** Patient data at PRA/IRA calculation time.

PRA-Related Data			IRA-Related Data		
Age Years (SD)	60.08	(7.96)	History of Stage II, III, and IV Periodontitis nb (%)	65	(89.04%)
Smoking status			Periodontitis susceptibility		
Non-smoker nb (%)	36	(49.32%)	Stage II—Grade A/B nb (%)	13	(17.81%)
Former smoker nb (%)	20	(27.40%)	Stage III—Grade A/B nb (%)	50	(68.49%)
Smoker nb (%)	17	(23.29%)	Stage III—Grade C nb (%)	6	(8.22%)
Diabetes nb (%)	3	(4.11%)	Stage IV nb (%)	4	(5.48%)
Tooth loss nb (SD)	8.44	(4.24)	Compliance level		
% BOP (SD)	24.29	(21.25)	Compliant nb (%)	61	(83.56%)
PPD ≥ 5 mm nb (SD)	7.85	(11.41)	Non-compliant nb (%)	12	(16.43%)
BL/age (SD)	0.59	(0.23)	RM-bone		
			Tissue-level implant nb (%)	70	(95.89%)
			Bone-level implant nb (%)	3	(4.11%)
			Prosthesis/plaque score		
			Less than 4 sites with PI > 1 nb (%)	48	(65.75%)
			Poor fit—supramucosal nb (%)	22	(30.14%)
			More than 3 sites with PI > 1 nb (%)	3	(4.11%)

**Table 4:** Patient risk score distributions.

PRA		
Low-risk nb (%)	8	(10.96%)
Moderate-risk nb (%)	38	(52.05%)
High-risk nb (%)	27	(36.99%)
IRA		
Low-risk nb (%)	0	(0.00%)
Moderate-risk nb (%)	53	(72.6%)
High-risk nb (%)	20	(27.39%)

**Table 5:** Associations between parameters, risk scores, and peri-implantitis incidence.

	Peri-Implantitis		
	*p*-Value	HR	CI (95%)
Tooth loss > 8	0.067	3.073	(0.924, 10.222)
BL/age > 1	0.003	6.134	(1.819, 100)
High Periodontitis susceptibility	0.071	3.086	(0.907, 10.526)
High-risk PRA	0.010	4.782	(1.46, 15.657)
High-risk IRA	0.038	3.653	(1.075, 12.416)

## Data Availability

The data presented in this study are available on request from the corresponding author.
